# Overview on Clinical Relevance of Intra-Tumor Heterogeneity

**DOI:** 10.3389/fmed.2018.00085

**Published:** 2018-04-06

**Authors:** Giorgio Stanta, Serena Bonin

**Affiliations:** DSM, Department of Medical Sciences, University of Trieste, Trieste, Italy

**Keywords:** intra-tumor heterogeneity, morphohistological intra-tumor heterogeneity, clonal intra-tumor heterogeneity, functional phenotypic plasticity, stochastic plasticity, cancer spreading, genomic instability

## Abstract

Today, clinical evaluation of tumor heterogeneity is an emergent issue to improve clinical oncology. In particular, intra-tumor heterogeneity (ITH) is closely related to cancer progression, resistance to therapy, and recurrences. It is interconnected with complex molecular mechanisms including spatial and temporal phenomena, which are often peculiar for every single patient. This review tries to describe all the types of ITH including morphohistological ITH, and at the molecular level clonal ITH derived from genomic instability and nonclonal ITH derived from microenvironment interaction. It is important to consider the different types of ITH as a whole for any patient to investigate on cancer progression, prognosis, and treatment opportunities. From a practical point of view, analytical methods that are widely accessible today, or will be in the near future, are evaluated to investigate the complex pattern of ITH in a reproducible way for a clinical application.

## Background

Today, the knowledge and the clinical evaluation of tumor heterogeneity are extremely important to improve clinical oncology. Inter-tumor heterogeneity exceeds the boundaries of specific tumors and also of their molecular classifications (1, 2), which makes the clinical approach very complex. However, the most complex issue is intra-tumor heterogeneity (ITH) as a spatial and temporal phenomenon more or less distinct in every single patient. This is closely related with cancer progression, resistance to therapy, and recurrences. Because of ITH in primary tumors and metastases, and because of the wide clinical heterogeneity among patients, it is necessary to apply clinical research methods directly to patients’ material in today’s clinical practice to be able to better define a specific effective treatment. For any type of tumor, only few molecular biomarkers are being used in diagnostics at the moment, and a minor part of available treatment targets is applied. Hopefully, wider clinical research directly performed on patients will be increasingly diffused as a requirement in the near future, with the goal to obtain more efficient and personalized therapy protocols (3). Moreover, phase three clinical trials in oncology have recently encountered wide criticisms (4–6), because of the long time, the high cost, and not always satisfying results. They are essentially based on a patient’s randomization process, which is unable to cover the entire range of clinical heterogeneity. Sophisticated methods of analysis allow penetration in the very high complexity of cancer biology; in clinical research, those methods can be directly applied on the patient to cover variability in toto. Therefore, only a wide clinical application of research methods can lead to a correct interpretation of the clinical reality. New types of clinical research approaches that consider heterogeneity have recently been suggested in oncology. Those approaches, such as N1 trials, basket, umbrella, and platform studies, have been proposed to overcome the limits of classical clinical trials and to shorten the time for a wide clinical application (7, 8).

**Figure 1:**
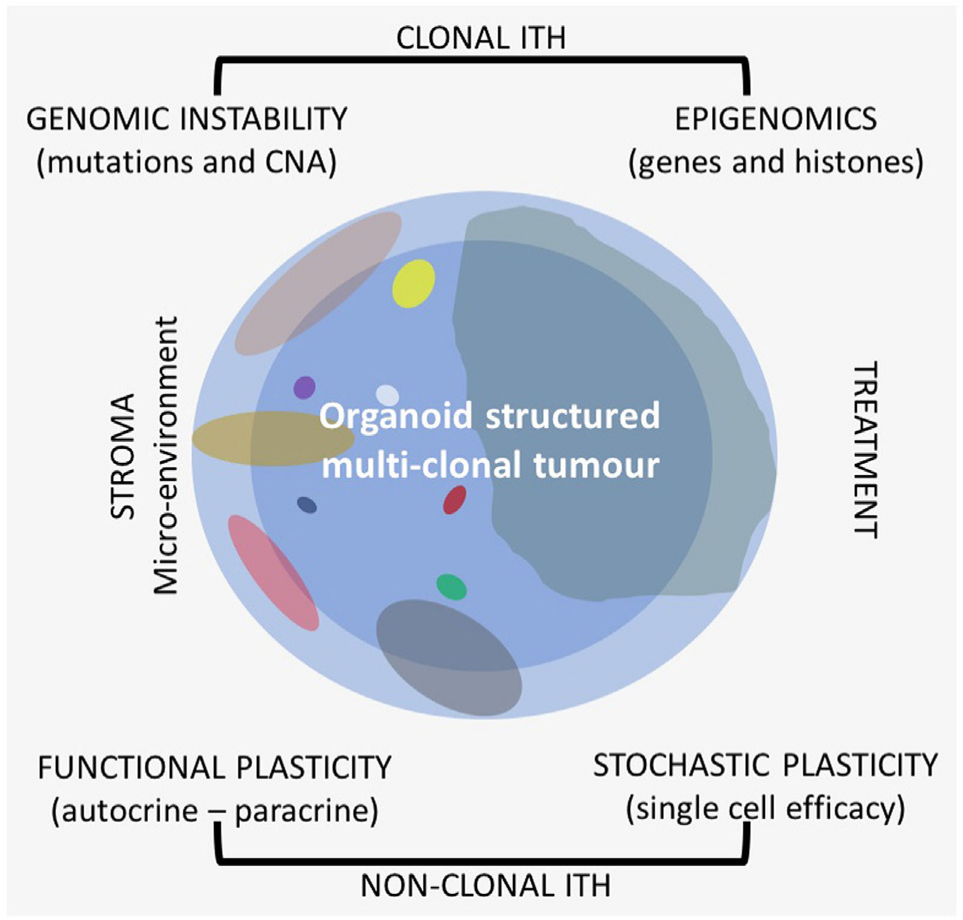
Different types of intra-tumor heterogeneity (ITH) in an organoid structured multiclonal tumor: the primary clone is blue (the peripheral area is light blue and the central one is darker), the other clones are of different size and multicolor.

In most papers, when it is reported about ITH, it is mostly dealt with clonal genetic evolution only, but ITH itself is a very complex matter because it is related to different sources and shows different patterns. Recently, also heterogeneity of drug distribution has been shown to be relevant in cancer treatment (9). There are several types of ITHs that can be observed on the morphological–histological and molecular level (Figure 1). All these should be taken into consideration when research is applied to clinics, because each patient needs to be considered as a whole also from this point of view for more effective and reproducible analyses.

On the morphological level, different histological heterogeneity patterns with different levels of differentiation are frequently observed in the same tumor, and it is well known that apart areas of the same tumor can have different patterns of gene expression also without any clonal evolution, e.g., the central part of the tumor compared to the external border (10). If these aspects of ITH are ignored, this can affect the reproducibility of clinical analyses and misinterpretation of the results.

On the molecular level, it is possible to distinguish at least two large categories of ITH, one of which is mostly clonal, transmitted to the daughter cells, and the other one is functional nonclonal (11–13). Recent literature reports have especially focused on the genetic clonal evolution of tumors based on DNA mutations and copy number alterations (CNAs). There is a lower extent of information on epigenetic evolution, which is also mostly clonal. Gene promoter methylation, general hypomethylation of tumor DNA, and histone methylation and deacetylation are very common in cancer and are as relevant as genetic alterations (14). The interaction between clonal genomic instability of cancer and the microenvironment leads to a nonclonal phenotypical functional plasticity which is related to autocrine and paracrine interactions with a quite wide phenotype range. Besides phenotypical functional plasticity, ITH is also related to a stochastic type of plasticity that can affect any single cell. Even in cell lines, each cell is different from the others with respect to efficiency and efficacy of the single cell machinery with variable time and level of gene expression.

Methodologically, to study patients, two separate phases should be distinguished: one should include more or less localized tumors for which surgical treatment is possible; the second one should involve advanced cancer and/or cancer recurrences. In the first case, a high level of molecular information to perform an effective adjuvant therapy to avoid recurrences is needed. This should be the main strategy to reduce cancer mortality together with early diagnosis procedures. The related clinical information is obtained from the analysis of primary tumor tissues: DNA, RNA, and proteins can be analyzed using extractive as well as *in situ* methods. The molecular methods that are applied by maintaining the morphology, such as immunohistochemistry (IHC) and/or *in situ* hybridization (ISH), can be specifically useful to study heterogeneity, preserving microenvironment interactions and to evaluate new types of therapy, such as immunotherapy (15).

As for recurrences, nowadays, an important tool, the “liquid biopsy,” is available. Cell-free plasma DNA (ctDNA) can be a useful instrument to analyze in those cases, giving important information for a more tailored treatment (16). New research methods and a closer clinical application are also appealing for circulating tumor cells (CTCs). This type of analysis gives further information especially on heterogeneity (17).

Intra-tumor heterogeneity analysis is the key for more efficient treatments of cancer in our patients, but only if highly reproducible clinical research is performed. We should consider that different aspects of ITH could be themselves one of the major sources of research irreproducibility, together with preclinical conditions of the biological materials and standardization of the analytical methods (11, 12).

## Morpho-Histological ITH

Quite often, different histological patterns are present in cancer, which are related to different levels of differentiation and to metaplastic changes. It was shown in lung cancer that the different histological patterns of the same tumor can correlate with different molecular alterations (18). Even the same histotype of tumor can have several levels of differentiation, related to a different grade of atypical cells that is measured by nuclear variation and other characteristics. A good example of a standardized analysis of cancer histological heterogeneity is prostate cancer. The Gleason score enables to measure the different levels of differentiation in a tumor and can give a specific grade, which is closely related to prognosis (19).

There is a relationship between histological pattern and molecular alterations. It was shown that in lung adenocarcinomas, the mutant allele frequencies were higher in solid areas of the same tumor (18).

**Figure 2:**
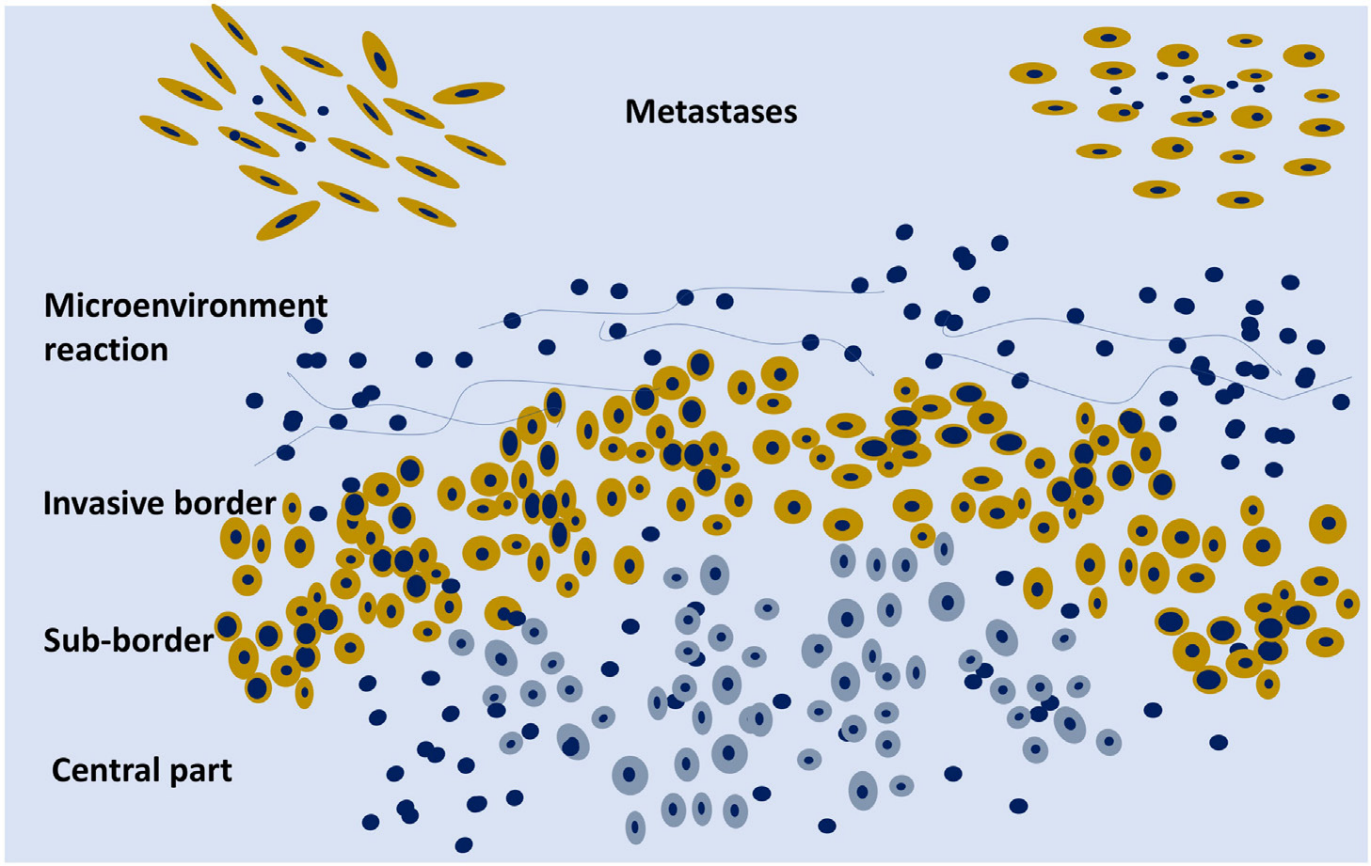
Schematic representation of an organoid tumor with peripheral cells (yellow) surrounded by stroma components and phenotypically different from the central cells (gray). On the upper part, two metastases are schematically represented.

The other important issue for the morphological pattern of a tumor is to consider distinct areas of the same tumor. Sampling from the border of the tumor, including the surrounding stroma and the sub-border in comparison with the central part of the tumor, can give distinctive information (Figures 1 and 2). Often, the border has a higher level of cellularity and higher neo-angiogenesis than the central part, and it is well known that gene expression differs in central or periphery location (10). At present, the tissue area of the tumor submitted to molecular analysis is hardly ever disclosed in the reports, and this could be one of the major issues referred to the low level of reproducibility not only of medicobiological research but also of diagnostics. Specific and standardized types of multiple sampling in larger tumors and microdissection in multiple sites in smaller tumors are the main tools that can improve reproducibility of molecular analysis in solid tumors, also taking ITH into account.

## Clonal ITH

For many years, it has been reported that tumor progression is associated with clonal evolution of the tumor cells (20). More recently, it has been clearly shown that this is the case in cancer (21). Genetic clonal evolution of cancer is related to genomic instability as a major feature of the carcinogenetic process. In recent literature, a wide description of specific genetic mechanisms involved in DNA instability has been documented. There is evidence on increased altered DNA replication when polymerases E or D are mutated (Pol-E) (22), or mismatched repair genes are mutated, or their promoters are methylated [microsatellite instability (MSI)] (23), or a chromosomal instability (CIN) with CNAs is present (24–26). Those mechanisms, once described in specific tumor types, are now reported to be differently related to progression and prognosis in several tumors, such as in cancer of endometrium, colon, pancreas, breast, lung, prostate, and kidney. Pol-E and MSI are connected with high ITH associated with a high number of DNA mutations (called ultra-mutated and hypermutated tumors, respectively) and with infiltration of tumor lymphocytes (TIL) due to the presence of a multitude of neo-antigens (27). Those tumors are associated with a better prognosis, especially for Pol-E. Neo-antigens are not only related to driver mutations but also related to passenger mutations, showing that also the latest are directly involved in cancer progression. On the other hand, a high level of CNA is also related to ITH, but in this case, TILs are rarer and the prognosis tends to be worse (27–34). Recently, it has also been shown in pancreatic cancer that CNAs increase in number from primary tumor to local lymph nodes, to distant metastases (35).

Clonal cancer evolution is related not only to DNA structural alterations involving protein coding genes but also to epigenetic mechanisms that are at the basis for altered gene expression. Noncoding gene alterations can be drivers in cancer progression (36, 37). Hypomethylation of DNA in cancer cells can trigger cancer-germline genes with their activation (38) and the hypomethylation of intra-genomic endo-parasitic DNA repeats such as L1 with their reactivation and retrotransposition can increase genomic instability (39, 40). L1 hypomethylation is present in NSCLC already in stage I tumors (41) and is related to worse survival in colon cancer stage II (42).

It is well known that tumor-suppressor genes can be methylated at the promoter level with loss of gene expression as one of the basic mechanisms of tumor progression, and this alteration can be transmitted to the next cell generations (43, 44). Methylation patterns in lung adenocarcinomas are present with higher ITH as compared with somatic mutation in the same tumors, suggesting to be later events in cancer progression (45). A CpG island methylator phenotype first described in colorectal cancer (46) was then reported in many different types of cancer as a generic disruption to epigenetic regulation (47, 48). Recently, it was shown that genome-wide methylation analysis can define pancreatic cancer subtypes (49).

Also, on the protein level, histone modification through methylation, deacetylation, phosphorylation, and ubiquitination can again silence tumor-suppressor genes or activate oncogenes (50–52). A new pathway was discovered in PTEN-deficient breast and prostate cancers with methylation of histone H3K4me3 that activates TNFa/NF-kB (53).

**Figure 3:**
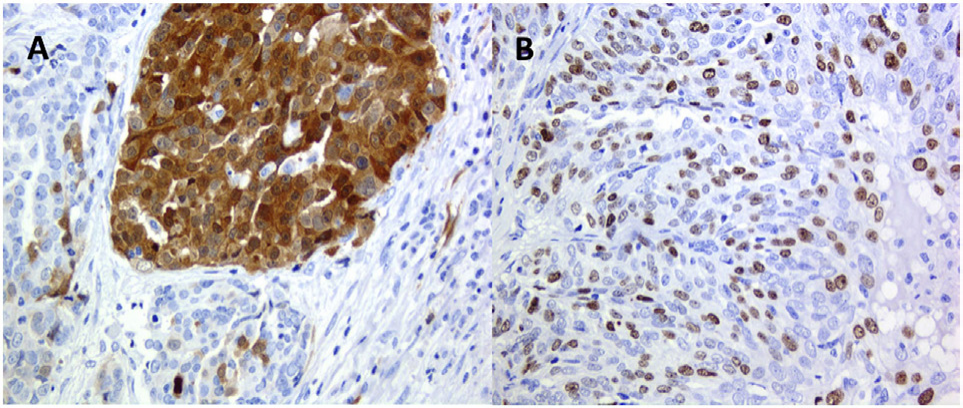
High-grade ovary serus carcinoma: **(A)** p16 clonal type intra-tumor heterogeneity (ITH) and **(B)** Ki67 stochastic plasticity.

All the clonal alterations can be spatial—within the primary tumor (Figures 2 and 3A), sometimes involving few cells with difficulties for the specific detection—and temporal, with the acquisition of new alterations (54). They can be defined as ubiquitous or truncal when, as driver mutations, they are involved in the carcinogenetic process from the initial phases, or shared, when they are shared by several different clones, and private, when they are specific of a single clone (21).

Clonal evolution is closely related to the applied cancer treatments; ITH is connected with biological therapy resistance by inducing the development of minor resistant clones (55, 56). Cytotoxic therapy also influences ITH with the risk to select more aggressive clones (57, 58).

## Nonclonal ITH

One of the major sources of functional gene expression ITH is related to the interaction of cancer cells with the microenvironment (Figure 1). There is an autocrine interaction within the same clone cells, among different clones in a synergistic and antagonistic way (59–61), and a paracrine interaction with stroma components. All are related to clonal evolution and tumor progression (62). The microenvironment influences cell phenotype in any type of clones; the interaction can vary from area to area of the primary tumor, and the stroma of the tumor could be heterogeneous itself (63–67). All these complex interactions are known as functional phenotypic plasticity, which is well recognized by pathologists that study, e.g., epithelial–mesenchymal transition (EMT) or as stemness of cancer cells (68). Intermediate expression patterns of cells in a different functional status can be recognized with the use of specific biomarkers showing the continuous evolving functional plasticity in cancer (69). There is not only the paracrine influence among cancer and stroma cells but also some kind of interaction with the different collagen types of the stroma that can also have an impact on treatment outcomes (70).

Clonal genetic alterations and the microenvironment influence each other: DNA alterations are permissive for the growth of cancer cells, but the microenvironment may favor the development of specific clones in comparison with others and the microenvironment may change in time.

In thyroid cancer, the close association of EMT and cancer stem-like cells (CSCs) as well as the role of exosomes and of the microenvironment in the metastatic phenotype were shown (71).

A crucial selection is driven by the specific microenvironment induced by clinical treatments. The selection and the development of new prevalent clones are likely to be driven by clones resistant to therapy, and the new treatment associated with the microenvironment drives the clonal genetic evolution. This gives an idea of how the different clonal and nonclonal interactions are closely related in the clinical progression and in a specific way for any single patient. Recently, it has been suggested that tumors should be classified in a different way considering ITH and its development. Tumors can be characterized by the level of clonal evolution over time and its interaction with the resources available at the microenvironment level (72).

A common phenomenon recognized, e.g., in immune-histochemical analysis, is that positivity for a specific antigen varies from cell to cell in the same tumor and in the same area of the tumor, from negative cells to highly positive (Figure 3B). This is related to another type of nonclonal ITH, the so-called stochastic plasticity (11–13). Each cell even in a cell line in culture has different efficacy on the transcriptional and translational level (73), and chaperone proteins such as Hsp90 may modulate phenotypic response. As a consequence, any phenotypic expression of a normal or an altered genome is further modulated differently in every single cell (74). Stochastic plasticity has been recently shown to influence resistance to chemotherapy modulating the dynamics of key players in response to treatment (75). DNA damage-inducing agents increase the expression of p53 and at the same time of inhibitor of apoptosis proteins, and resistance to chemotherapy can be caused by stochastic fluctuations in protein levels (75).

Cancer should not be considered just as an agglomerate of cells, but as a functional organoid structure: the central part of the tumor can be hypoxic and the border well oxygenated with molecular metabolic exchanges between the two parts, e.g., lactate produced by the hypoxic cells can be metabolized on the mitochondrial level by the activity of lactate dehydrogenase in the peripheral cells with a higher level of oxygenation (Figure 2). There is an intra-tumor metabolic heterogeneity with different pathway activation, which has recently been defined in kidney cancer (76). Tumor hypoxia influences not only the evolution of cancer cells but also the development of the stromal microenvironment (77).

## ITH in Metastatic Spread

Intra-tumor heterogeneity usually refers to intra-primary-tumor heterogeneity, but it is also related to inter- and intra-metastatic heterogeneity. There are several reports in literature denying significant differences between primary tumors and metastases, but this is sometimes due to an inadequate number of analyzed samples as well as to the limited number of used biomarkers (26, 78). Clonal selection is the main mechanism underpinning those differences (79); therefore, it easily explains the reduced intra-metastatic heterogeneity reported (80). More aggressive and lethal clones can arise from minor subclones of the primary tumor even of relatively low grade (81, 82).

Monoclonal seeding of metastases has recently been dueled, suggesting the possibility of polyclonal seeding (80). Inter-metastatic exchange of cancer clones that evidence the potentiality of metastatic sites to act as a primary tumor was also detected (83), and this can raise relevant considerations in favor of surgical treatment of the major metastases.

A recent work has shown that in most cases of colon cancer, the subclonal origin of the local lymph-node metastases is different from the distant metastases in other organs, denying the possibility that the latest can derive from the metastatic lymph nodes as often suggested (84).

The microenvironment differs between primary tumor and metastases influencing the phenotype of tumor cells (85). Usually, clinical biomarkers are only searched for in primary tumors, although treatments and outcomes could be better defined by their analysis in the metastatic tissues (86). Furthermore, by comparing the expression of biomarkers’ profile between primary and metastasis, the molecular classification of breast cancer can vary during cancer spreading in comparison with the primitive site. Clinical relevance of a specific biomarker has also been shown to vary if the biomarker was detected in the primary tumor or in the lymph-node metastasis (87).

## Methods to Study ITH

### Sampling

Fixed (mostly in formalin) and paraffin-embedded (FFPE) tissues are the main resource of clinical tissue, and they are the only tissues available for any patient. The methods reported hereafter are those adapted to that type of tissues.

Most genes are not involved in clonal evolution, so it is possible to obtain reproducible quantitative analysis on the mRNA level comparing multiple samples of the same tumor taken from similar tumor areas, such as the infiltration sub-border. However, this is not the case, with an important difference in gene expression, if a specific microdissected area of the tumor is compared with the analysis of the entire tumor (12). To that purpose, accurate microdissection is required for analyses. Microdissection must avoid residual normal tissues that can modify the results of the analysis, as it is shown for several prognostic clinical signatures in breast cancer. The risk category changed along with the quantity of normal gland present in the analyzed tissue (88). An accurate sampling and analysis of primary tumors can give critical clinical information also about stroma and immune-score characteristics, as it has been recently shown in colon cancer (89). For analysis of macromolecules, microdissection is essential for comparable results and the microdissected area should be described in the report.

**Figure 4:**
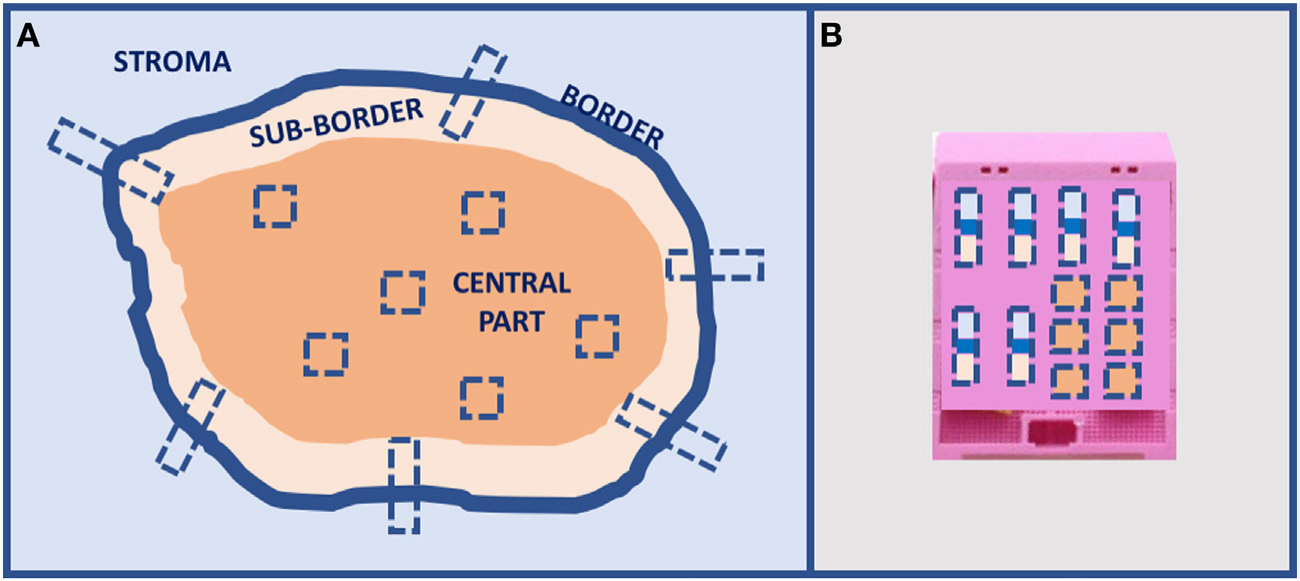
Proposal of tumors larger than 2-cm sampling for *in situ* analyses: **(A)** multiple sampling locations and **(B)** organization in the inclusion block.

A preliminary type of sampling standardization has been proposed for solid tumors larger than 2 cm for IHC and ISH analyses, and it is going to be tested in the most common tumors for a large number of cases using the current clinical immune-histochemical biomarkers (Figure 4). This procedure has been proposed for a multicentric study involving different working groups of the European Society of Pathology. Today, there is an urgent necessity to standardize sampling procedures in light of the hugely confusing data reported in the scientific literature about frequency of ITH in any type of tumor.

### Extractive Methods

Nowadays, nucleic acids extraction from clinical tissues can be a reproducible practice if it is standardized and checked with specific controls for FFPE tissues (90, 91). For clinical research activities, also samples fixed with Bouin’s solution, which has a higher nucleic acid level of degradation, can be used with specific precautions (92). To check the final progression of cancer, also autopsy tissues can be used in many cases (93–95).

Next-generation sequencing (NGS) sensitivity is high enough to detect most clonal DNA alterations. Whole-exome sequencing (WES) of DNA in clinical tissues of primary tumors can detect several alterations present in the tumor clones that could be useful for treatments. Actionable mutations could suggest the use of specific biological drugs for that specific tumor. Actionable mutations have been detected in 90% of patients by WES (mean 4.9 per patient), a 7.5-fold, a 2.0-fold, and a 1.9-fold increase over the amount obtained by using the most common NGS gene panels, namely CHPv2, OCP, and FoundationOne, respectively (96). Seventy percent of the patients have an actionable alteration with the availability of an approved drug, but in only 20%, the drugs were approved for that specific type of tumor (on-label) (97). Recently, it has also been shown that not only non-synonymous mutations are involved in drug response or resistance but also synonymous ones, e.g., in response to anti-EGFR therapy in colon cancer and head and neck squamous carcinomas (98, 99). These figures show the importance of clinical research directly applied to patients and the necessity of new types of clinical studies.

Specific attention should be given not only to a correct technology in NGS but also to a careful interpretation and to the presence of artifacts due to fixation, such as false transition mutations (100).

DNA and RNA single cell sequencing have already been developed, and in case of DNA, it can give important information on ITH (101–103), but we should be very careful in the interpretation of RNA data because the different level of expression in every single cell could be due to stochastic plasticity.

Several methods have been used to study epigenetics in FFPE tissues. For a genome-wide methylation analysis, it is possible to use NGS-related methods or microarrays in a representative tumor sample or in different microdissected tumor areas (104), but also other specific methods, such as methylation-specific multiple ligation-dependent probe amplification, have been used (105). To quantify all the four known DNA-methylated derivatives of cytosine, namely 5 methylC, 5 hydoxymethylC, 5 formylC, and 5 carmoxylC, in FFPE tumor samples, liquid chromatography/mass spectrometry methods have been developed (104). Although it is less precise than LC/MS methods, also ELISA-based immunoquantification technology is available to detect the abovementioned methylated derivatives of cytosine in a simpler way (106).

### Liquid Biopsy

The term “liquid biopsy” was originally introduced for CTCs, recovered from (liquid) peripheral blood (17). This method has rapidly improved to be shortly used also on the clinical level, the low number of collected cells being the major limit. Recently, circulating clusters of cells instead of single CTCs have been shown in breast cancer patients, which are related to higher spreading, worse prognosis, and chemo-resistance (107). This evidence could explain the presence of polyclonal seeding in the cancer metastatic process.

Liquid biopsies do not only contain CTCs but also cell-free nucleic acids and exosomes. For the presence of tumor-specific (somatic) variations in cancers, the fraction of circulating cell-free plasma tumor DNA (ctDNA), together with the larger amount of circulating cell-free DNA from normal cells, can be used as specific blood-based analyses for cancer patients’ care. Thus, the use of a patient’s “liquid biopsy” can allow identifying residual micro-metastatic cancer and investigating specific mutations without any invasive intervention (16). “Liquid biopsy” can theoretically offer a real-time assessment of molecular tumor genotype (qualitatively) and existing tumor burden (quantitatively) and in this way also ITH information. The major limitation for plasma tumor DNA has been the low detection rate; however, new techniques, such as digital PCR, have increased sensitivity (16). The ideal assays for liquid biopsies ctDNA would allow interrogating mutations in several genes at the same time, which represents a technical challenge because the total amount of ctDNA and its quality are both relatively low (56). The analysis of ctDNA can be helpful during the follow-up of patients to detect both truncal and private mutations. However, there is the need to better understand the contribution of each dynamic cell population in a heterogeneous tumor to ctDNA (56). It is manifest that CTCs and ctDNA can come from different and heterogeneous metastatic sites; therefore, sensitivity and reproducibility in detecting tumor range still have to be established (108, 109). Furthermore, in the scenario of tumor heterogeneity it should be highlighted that these low invasive methods that can be repeated many times during the follow-up, are especially useful, because the same cancer therapy can drive the selection pressure that causes clonal evolution (55).

### CRISPR Barcoding in Heterogeneity

Detection of preexisting resistant subclones could be hard from a methodological point of view, because of their rarity. Sensitivity of the current methods is not enough to comprehensively consider cancer individual cells in heterogeneous cancer-cell populations (110). Recently, Guernet et al. developed a highly sophisticated CRISPR-barcoding system that enables the functional investigation of specific mutations, in a context that closely mimics the complexity of cancer (111). The high-resolution tracking of single specific cancer cells allows identifying even rare preexisting resistant subclones that can be involved in acquired resistance to therapy. Using CRISPR/Cas9 technology, the fastest ever genome engineering technology, and specific DNA barcodes, a strategy to recapitulate and trace the emergence of subpopulations of cancer cells containing a mutation of interest has been developed. The method has already been used to study mechanisms of lung cancer cell resistance to EGFR inhibitors and to investigate on combined drug therapies. Highly complex barcodes inserted in a specific genome location have been used to simultaneously trace the fates of many thousands of genetically labeled cancer cells (111). This methodology could significantly improve the understanding of ITH and its relationship with tumor progression.

### Molecular Morphology

The complexity of ITH is strictly related to the biology of the tumor, to treatment possibilities, and to recurrence probability. As a consequence, we need more sophisticated, sensitive, and morphology-related methods that can provide information on different clones or different cell types at the molecular level and their relationship with the microenvironment. Localized complex phenomena, such as tumor budding, can be studied only by morphology-related methods that show close localized relationship between cancer cells and stroma (65). ITH is indeed detectable not only among cancer cells but also at the tumor stroma level (112). Recently, molecular morphology has demonstrated its utility in predicting the efficacy of immunotherapy (15).

IHC for proteins and ISH for DNA and RNA can be successfully used in a reproducible way in FFPE tissues. The interplay of these methods in digital analysis is going to gain a more objective quantitative evaluation.

FISH (and other similar methods) for DNA and IHC for proteins have been performed for many years also at the clinical level, but today, we also have reproducible *in situ* methods for mRNAs and noncoding RNAs and developing tools to detect most genetic, genomic, and epigenetic types of alterations. ITH can be studied at the *in situ* level for gene amplification and CIN with FISH (113, 114), for single nucleotide mutation (115), for fusion transcripts detection (116), for repetitive RNAs related to hypomethylation (117), and for *in situ* detection of histone modification (118). For proteins, more sophisticated methods, such as matrix-assisted laser desorption/ionization imaging can be used (119). Most of those *in situ* techniques are not very widely diffused as methods for clinical research, and pathologists should improve their experience in this field.

Pre-analytical conditions of tissues are a basic prerequisite for reproducible results even for *in situ* methods. Technical CEN specifications are already available for extractive methods in FFPE tissues (DNA, RNA, and proteins) and for ctDNA from plasma as supported by SPIDIA4P EU project^1^ and developed by the European Committee for Standardization.^2^ For *in situ* methods, ISO documents are ongoing as described in SPIDIA4P EU project.

## Conclusion

There is the necessity to consider ITH from a practical point of view to improve diagnosis and treatment in cancer. The major remarks could be the following:

– Clinical tumor heterogeneity on the inter- or intra-tumor level limits the utility and the application of tumor molecular classifications based on few molecular common biomarkers.
– There is an increasing necessity to apply clinical research directly to clinics, a research performed for the today’s patients with new types of studies to shorten the time for wide clinical application.
– ITH in this type of research is crucial, because it is closely related to cancer progression, therapy resistance, and recurrences.
– ITH shows different aspects in cancer, on the morphological–histological level, and on the molecular one. Molecular ITH can be divided into clonal and nonclonal. The clonal one is related to different types of genomic instability that also influence aggressiveness and treatment. The nonclonal one is functional, microenvironment related, or stochastic, notably single cell efficiency related. Clonal and microenvironment ITHs are closely connected and influence each other.
– All these types of ITH affect cancer progression and treatment efficacy and should be considered as a whole for any patient.
– At least two types of ITH methodological approaches should be considered: in surgically treated tumors, a careful analysis of tissues should drive the adjuvant therapy, and in recurrent cancer, the follow-up should consider the inclusion of blood analysis of ctDNA and, in a near future, also CTCs.
– Actionable mutations and resistance alterations should also be detected in minor clones to establish a better and tailored treatment.
– For tissues, multiple microdissection of tissues should be performed, and for larger tumors, a standardized multiple sampling procedure should be adopted to increase analysis reproducibility.
– Pathologists should improve their experience in *in situ* methods to better study clonal and microenvironment interactions.

## Author Contributions

All co-authors of this manuscript contributed for conception or design of the work (mostly GS), drafting the work or revising it critically for important intellectual content, and final approval of the version to be published.

## Conflict of Interest Statement

The authors declare that the research was conducted in the absence of any commercial or financial relationships that could be construed as a potential conflict of interest.

## Footnotes

^1^Standardisation and improvement of generic pre-analytical tools and procedures for in-vitro diagnostics (SPIDIA and SPIDIA4P). Available from: http://www.spidia.eu/about-the-projects/.

^2^European Committee for Standardization (CEN). Available from https://www.cen.eu/Pages/default.aspx.
